# Identification of Seven Aberrantly Methylated and Expressed Genes in Adrenocortical Carcinoma

**DOI:** 10.3389/fendo.2019.00472

**Published:** 2019-07-12

**Authors:** He Xiao, Weixiang He, Ping Chen, Deqiang Xu, Guang Zeng, Zhuo Li, Mingliu Huang, Xinghuan Wang, Michael E. DiSanto, Xinhua Zhang

**Affiliations:** ^1^Department of Urology, Zhongnan Hospital of Wuhan University, Wuhan, China; ^2^Department of Urology, Shenzhen Sixth People's Hospital, Shenzhen, China; ^3^The Sixth Affiliated Hospital of Shenzhen University Health Science Center, Shenzhen, China; ^4^Affiliated Shenzhen Sixth Hospital of Guangdong Medical University, Shenzhen, China; ^5^Shenzhen Key Laboratory for Endogenous Infection, Shenzhen, China; ^6^Departments of Biomedical Sciences & Surgery of Cooper Medical School of Rowan University, Camden, NJ, United States

**Keywords:** adrenocortical carcinoma, bio-informatics, methylation, differentially expressed genes, biomarkers

## Abstract

**Background:** Adrenocortical carcinoma (ACC) is a rare endocrine malignancy with an unfavorable prognosis and limited treatment options. Nevertheless, no clinically applicable molecular markers have been identified for the progression of ACCs. DNA methylation alterations were found to contribute to the development of ACC in recent decades.

**Material and Methods:** The aims of the current study was to identify the abnormally methylated differentially expressed genes (DEGs) in ACCs, and to elucidate the mechanistic basis for these changes. Analyses were conducted on gene expression and gene methylation profile datasets to identify the aberrantly methylated DEGs. The DAVID software was used to conduct the analyses of functional enrichment on screened genes. Finally, expression was validated, and the relationship between abnormally methylated DEGs and clinical features was determined via the Oncomine database and The Cancer Genome Atlas (TCGA). To further verify the altered expression and methylation status of our identified genes we also validated these changes at the tissue and cellular levels.

**Results:** We screened and identified 92 differentially expressed genes and 802 abnormally methylated genes. Furthermore, seven aberrantly methylated and dysregulated genes were identified and validated, along with a number of functional enriched pathways. Among these seven genes, the expression or methylation status is significantly correlated with different pathological stages and overall rates of survival. In validation, the expression of seven genes were significantly altered and five genes were hypermethylated in ACC.

**Conclusions:** Our study identified abnormally methylated DEGs and potentially affected pathways in ACCs, from which we could begin to understand the basic molecular mechanisms of these alterations. Moreover, these abnormally methylated genes might serve as therapeutic targets and biomarkers to allow ACC patients to be more precisely diagnosed and effectively treated.

## Introduction

Adrenocortical tumors have a relatively high incidence of about 1–10%, most of which are benign tumors discovered by accident (1). In contrast, Adrenocortical carcinoma (ACC), is the most aggressive among all types of adrenal tumors and has a quite low incidence with only 0.7–2 diagnosed patients in one million people every year (2, 3). Nevertheless, this cancer is very deadly, yielding an average 5 year survival rate under 35% (4–7). ACCs are generally discovered in grown-ups aged 40–50 years, while it may also happen to children, especially those with a typical germline mutation in tumor protein p53 (8). Additionally, the metastasis of ACCs usually happens simultaneously when the cancer is diagnosed, resulting in poor prognosis (9). For the majority of the patients, mitotane treatment and systemic adjuvant chemotherapy are necessarily required to reduce the excessive secretion of hormones and the progression of the tumor (10–12). However, the effects of these agents are generally poor. At present, surgery has been taken as the only truly curative treatment for this disease. If the ACC tumor cannot be resected completely, the remaining therapeutic choices show little effect on the improvement of patient survival (13). In recent years, many studies identified genetic and epigenetic alterations in ACCs and suggested them to be key factors for the occurrence and development of ACCs. Hence, it is of great significance to unravel the alterations of genes in ACCs that can lead to the discovery of useful therapeutic targets along with an increased ability to predict individual responses.

Ever since the beginning of the post-genomic era, studies on tumorigenesis mechanisms have no longer been limited to gene deletion and mutation. In recent years, epigenetics has begun to play important roles in the study of tumorigenesis. DNA methylation is an epigenetic modification that is not accompanied by any alteration of DNA sequence, and usually occurs in the context of CpG dinucleotides. There are two types of DNA methylation patterns. One is a CpG-island hyper-methylation occurring in particular gene promoter areas, which is always related to tumor suppressor gene transcriptional silencing. Another is a global hypo-methylation related to increased instability of the chromosomes, reactivation of transposable elements, and parental imprinting loss (14). The abnormal DNA methylation can impact tumorigenesis and tumor progression by influencing the regulatory patterns of oncogenes and anti-oncogenes, as well as the structure of chromatin at the level of transcription (15, 16). Meanwhile, an increasing number of studies have been suggesting a critical role of histone modification and DNA methylation in the occurrence of tumors. Additionally, there is evidence showing the intimate relationship between tumorigenesis and GpG-island hyper-methylation which causes the silencing of downstream genes (17–19). With regard to adrenocortical tumors, evidence has shown the involvement of H19 promoter DNA methylation alteration in ACCs (20). Therefore, the abnormal methylation genes might be rediscovered as potential therapeutic targets or bio-markers for ACC.

In recent years, high-throughput sequencing has become a powerful tool which could search for epigenetic alternations in genes and provide more useful information for diagnosis and prognosis in oncology. Differentially expressed genes (DEGs) and differentially methylated genes (DMGs) between tumor samples and normal controls can both be obtained from microarray data via a high-throughput platform. This data could lead to the identification of important epigenetic targets in ACCs.

There are very few studies that have simultaneously examined gene-methylation-profiles and gene-expression-profiles and their link to ACC development. In the current study, bio-informatics was employed to conduct systematic analysis and integration of the data collected from a gene-methylation-profile microarray (GSE77871) and multiple gene-expression-profile microarrays (GSE75415, GSE90713, GSE12368, GSE14922, and GSE19750. The analysis process involved screening gene enrichment in pathways and gene ontology (GO), overlapping five datasets with the use of a Venn diagram, obtaining DMGs and DEGs with the use of R software and validating them in a TCGA database, Genotype Tissue Expression (GTEx) database, Oncomine database and a GSE49280 database. This current study thus, seeks to identify abnormally methylated DEGs, along with alterations in signaling pathways and GO among normal and tumor samples and using this information elucidate the molecular mechanisms of ACCs.

## Materials and Methods

### Microarray Data Information

Raw gene expression profiles (GSE75415, GSE90713, GSE12368, GSE14922, and GSE19750) and a raw gene methylation profile (GSE77871) were obtained from Gene Expression Omnibus (GEO) database (http://www.ncbi.nlm.nih.gov/geo/). Datasets GSE12368 (21) and GSE19750 (22) were performed on the same platform Affymetrix Human Genome U133 Plus 2.0 Array (HG U133 Plus 2.0). Dataset GSE14922 (23), GSE75415 (24), and GSE90713 (25) were performed on the platform Agilent-014850 Whole Human Genome Microarray 4x44K, Affymetrix Human Genome U133A Array and Affymetrix Human Gene Expression Array, respectively. These five datasets were combined and analyzed to screen DEGs. Dataset GSE77871 (26) based on the platform of Illumina HumanMethylation450 was used to screen DMGs. In addition, RNA sequencing data of 79 ACCs samples was downloaded from The Cancer Genome Atlas (TCGA) database (https://genome-cancer.ucsc.edu/) for further validation. Gene expression data were based on Illumina Hiseq's RNA sequencing technology. Six normal samples and 18 ACC samples were included in the data of the gene-methylation-profile microarray, while 26 normal samples and 132 ACC samples were included in the data of the gene-expression-profile microarray. In order to verify the association between gene methylation and expression, we also downloaded the GSE49280 data.

### Data Processing for the Identification of DEGs and DMGs

Raw expression data was calculated using preprocessing procedures: RMA background correction, log2 transformation, quantile normalization, and median polish algorithm summarization using the “affy” (27) package of R software (version 3.5.0; Bell Laboratories, formerly AT&T, now Lucent Technologies, Murray Hill, NJ, USA). Additionally, “sva” (28) R package was used to remove batch effects between five datasets. Probes were annotated by the Affymetrix annotation files. R package “limma” (29) was applied to select the DEGs between 132 ACC samples and 26 normal samples. The cut-off standards for DMGs and DEGs were β > 0.2, *p* <0.05 and |logFC| > 1, *p* <0.05, respectively. Subsequently, the wANNOVAR tool (http://wannovar.wglab.org/) was used to uncover the identities of DMGs. Then, the Venn diagram tool (http://bioinfogp.cnb.csic.es/tools/venny/) was applied to overlap the DEG identification processes for five gene-expression profile datasets. Finally, the upregulated hypo-methylated genes were acquired from the overlapped upregulated and hypo-methylated genes. The downregulated hyper-methylated genes were similarly acquired.

### Gene Ontology and Pathway Enrichment Analysis of DMGs

For the analysis of gene ontology enrichment, the gene list, which contained 471 hyper-methylated and 336 hypo-methylated genes, was submitted to the DAVID website https://david-d.ncifcrf.gov/. The GO outcome *p*-value-ranking was performed with *p* <0.01 as statistically significant. Those cut-off values of statistical significance were selected for molecular function (MF), cell component (CC), and biological processes (BP). For the analysis of KEGG (Kyoto Encyclopedia of Genes and Genomes) pathway enrichment, the cut-off standard was set as *p* < 0.05.

### Validation of the Identified Genes Expression and Methylation Levels

The multidimensional and comprehensive key genomic change maps for a variety of cancers were contained in the Oncomine, GTEx and TCGA databases. The identified genes were further validated with these three databases. Gene expression was compared between ACC and normal samples. In particular, TCGA data were analyzed with cBioPortal. As a publicly accessible platform, which provides the functions of downloading, analyzing, and visualizing of massive datasets of cancer genomes for different types of cancers, the tool of cBioPortal helped immensely in examining the correlation of DNA methylation with mRNA expression in the studies on ACCs, as well as for the altered genetic information associated with DMGs.

### Correlation of the Identified Genes Expression and Methylation With Pathological Stages

We downloaded the TCGA data from the Genomic Data Commons Data Portal (https://portal.gdc.cancer.gov/) which is a robust data-driven platform which provides the functionality of data searching and downloading for bioinformatics and cancer researchers. The boxplot was applied to show the identified genes' relationship with the different pathological stages. One-way ANOVA was used to determine whether the findings are statistically significant.

### ACC Cell Lines and Human ACC Samples

Human ACC cell lines (SW13, NCI-H296R) were purchased from the Procell Life Science & Technology Co., Ltd. in Wuhan, China. The cell line was identified by the China Centre for Type Culture Collection in Wuhan, China. SW13 was incubated in DMEM medium (Gibco, China), supplemented with 10% fetal bovine serum (FBS) (Gibco, Australia) according to the ATCC instructions. NCI-H296R was grown and maintained in DMEM media supplemented with 1% insulin transferrin selenium (Gibco, China) and 2.5% Nu-Serum I (Gibco, China) in a standard humidified incubator at 37°C in a 5% CO_2_ atmosphere. ACC and adjacent normal adrenal tissues (*n* = 8) were obtained from patients undergoing laparoscopic unilateral adrenalectomy at Zhongnan Hospital of Wuhan University. Two pathologists independently confirmed the histological diagnosis. All specimens were immediately fixed in 4% PFA (paraformaldehyde) and stored in liquid nitrogen. Prior to their operation, none of the patients enrolled in the study receive any treatment. The use of these ACC specimens was approved by the Ethics Committee at Zhongnan Hospital of Wuhan University, and informed consent was obtained from all patients.

### Promoter Methylation Analysis and 5-Aza-2′-deoxycytidine Treatment

Bisulfite sequencing PCR (BSP) of the six methylated gene was accomplished by Sangon Biotech Co., Ltd. (Shanghai, China) and primers for the analysis were designed using the free on-line software MethPrimer (30). ACC cells were treated with 5-aza-2′-deoxycytidine (50 mM, Sigma-Aldrich, St. Louis, MO) for 48 h with a change of culture medium every day.

### Total RNA Extraction and Real-Time RT-PCR

Total RNA was isolated from the frozen tissues using Takara RNAiso Plus (Takara Bio. Inc., Otsu, Shiga, Japan) according to the manufacturer's protocol. Genomic DNA (gDNA) was removed and cDNA was reverse-transcribed using Takara PrimeScript^TM^ RT reagent Kit with gDNA Eraser (Takara Bio. Inc., Otsu, Shiga, Japan) in a T100^TM^ Thermal Cycler System (BioRad, USA). The experimental protocol utilized was first gDNA removal (42°C, 2 min), followed by reverse transcription (37°C 15 min, 85°C 5 s). Subsequently, all samples were amplified by a 25 μl reaction volume in a CFX96^TM^ Real-time PCR Detection System (BioRad, USA), using SYBR^®^ Premix Ex Taq^TM^ II (Takara Bio. Inc., Otsu, Shiga, Japan). All samples were run in triplicate. The seven identified genes were investigated. The amplification program was repeated for 40 cycles. Primer sequences are shown in Table 1. For relative quantification, gene expression was normalized to expression of *GAPDH* housekeeping gene and compared by 2^−ΔΔCT^ method.

**Table 1:** Primer sequence used for qPCR.

**Target Gene**		**Primer sequence**	
*NR2F1*	Forward	5′-CATTTTTGGGCGATCTCCAG-3′
	Reverse	5′-CTCGCTGCCTTCTTCTTTCG-3′
*KCNQ1*	Forward	5′-GTCCATGCAACAAGGTGGTC-3′
	Reverse	5′-GTTGTTCGTAGGTGGGCAGG-3′
*HOXA5*	Forward	5′-GCGTGGAAGTGTTCCTGTCT-3′
	Reverse	5′-ACCGCTTGGAGTCACAGTTT-3′
*CD14*	Forward	5′-AAGCACTTCCAGAGCCTGTC-3′
	Reverse	5′-TCGTCCAGCTCACAAGGTTC-3′
*PTGER4*	Forward	5′-GATCAAATGCCTCTTCTGCCG-3′
	Reverse	5′-ACTGCTGATCTCCTTCAGCTC-3′
*CYP11B1*	Forward	5′-TGATGGCTTTGGTGCATGTG-3′
	Reverse	5′-CCCAACCGTGATCTGTCTGT-3′
*ESM1*	Forward	5′-TTGCTACCGCACAGTCTCAG-3′
	Reverse	5′-GCAGGTCTCTCTGCAATCCA-3′
*GAPDH*	Forward	5′-ATCCCATCACCATCTTCCAGGAG-3′
	Reverse	5′-CCTGCTTCACCACCTTCTTGATG−3′

### Immunofluorescence

Tissues were sectioned in 10 μm thick slices and thawed, mounted onto glass slides using a cryostat (Leica CM 1850, Wetzlar, Germany), air-dried, and fixed for 10 min in ice cold acetone. Slides were washed in PBS and incubated for 2 h in a mixture of PBS supplemented with 0.2% Triton X-100 and 0.1% bovine serum albumin, followed by incubation overnight with the primary antibody (1:100). The secondary antibody employed to visualize the localization of HOXA5 is Cy3-conjugated goat anti-rabbit IgG (1:1,000). DAPI was used for staining the nucleus. Visualization was done with a laser scanning microscope (Olympus, Tokyo, Japan).

### Survival Analysis

The survival rate analysis was conducted based on the TCGA database for the assessment of the identified genes' effects on the prognosis of ACC patients. Firstly, patients with mRNA data were classified in two different categories in accordance with each gene's median expressions (low vs. high). Patients with methylation data were similarly analyzed. Secondly, analysis was conducted on the patients with both mRNA and methylation data. Finally, we performed Kaplan-Meier survival analysis and the log-rank test by adopting the “survival” R package. One-way analysis of variance (ANOVA) and paired 2-tailed Student's *t*-tests were used to analyze the statistical significance of differences of data.

## Results

### Identification of the DEGs and DMGs

**Figure 1:**
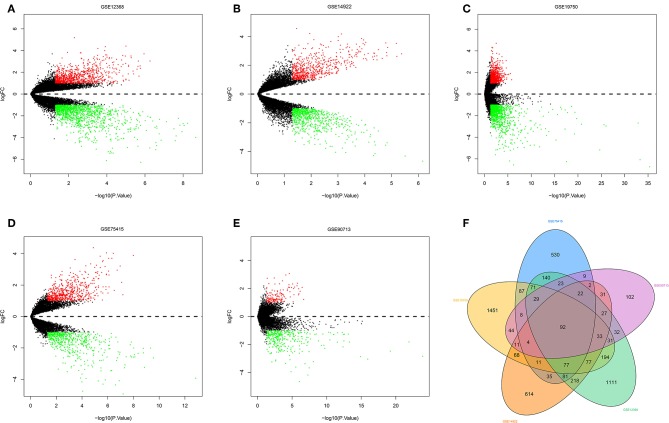
Differentially expressed genes and common differentially expressed genes in five datasets. **(A–E)** are volcano plots visualizing the differentially expressed genes in GSE12368, GSE14922, GSE19750, GSE75415, and GSE90713, respectively. The dotted line corresponds to log2 FC = 0. The red nodes represent upregulated genes with a log2 FC ≥ 1. The green nodes represent downregulated genes with a log2 FC ≤ 1. **(F)** Common differentially expressed genes in five datasets.

**Figure 2:**
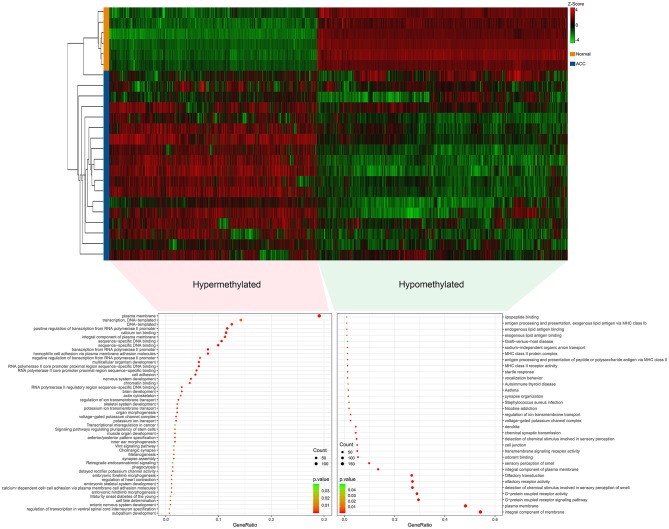
Differentially methylated genes (DMGs) in GSE77871 and functional enrichment of DMGs. Upper panels are heat maps of hierarchical clustering revealing 807 genes that were differentially methylated including 471 hypermethylated genes and 336 hypomethylated genes in ACC groups when compared with normal groups. Red and green colors indicate hypermethylation and hypomethylation, respectively. Lower panel is gene ontology and KEGG pathways enrichment ranked by *p*-value for the aberrantly methylated differentially expressed genes using the DAVID software. A *p* < 0.05 was regarded as significant.

The R software was used to preprocess data and assess quality. Then the matrices of expression were obtained from datasets of GSE75415, GSE90713, GSE12368, GSE14922, and GSE19750 (containing 132 ACC samples and 26 normal samples, Table 2). The DEGs of five datasets are shown as volcano plots in Figures 1A–E. We overlapped the DEGs of five datasets. In total we identified 92 DEGs with 57 downregulated and 35 upregulated genes in common between the 5 sets (Figure 1F). The DMGs are shown as a heatmap in Figure 2. In total we identified 802 DMGs including 336 hypo-methylated and 466 hyper-methylated genes in GSE77871.

**Table 2:** The detailed information of the six GEO datasets.

**Dataset**	**No. of samples (N/T)**	**Array types**	**Experiment type**	**Origin**
Soon et al. GSE12368	6/12	Affymetrix Human Genome U133 Plus 2.0 Array	mRNA	Endocrine-related cancer 2009/6/1
Tombol et al. GSE14922	4/4	Agilent-014850 Whole Human Genome Microarray 4x44K G4112F	mRNA	Endocrine-related cancer 2009/9/16
Demeure et al. GSE19750	4/44	Affymetrix Human Genome U133 Plus 2.0 Array	mRNA	Surgery 2013/6/28
West et al. GSE75415	7/15	Affymetrix Human Genome U133A Array	mRNA	Cancer Research 2007/1/15
Weiss et al. GSE90713	5/57	Affymetrix Human Gene Expression Array	mRNA	Oncotarget 2017/9/26
Legendre et al. GSE77871	6/18	Illumina HumanMethylation450	DNA	PLoS ONE 2016/3/10

### Analysis of Functional Pathway Enrichment for 802 DMGs

Gene ontology (GO) and KEGG analyses of hypo-methylated and hyper-methylated genes were displayed in Figure 2. GO analysis indicated 42 terms (biological process, cellular component and molecular function) enriched from 466 hyper-methylated genes (*p* < 0.01). Among those, 18 terms were found most significant (*p* < 0.001). KEGG analysis found seven pathways were significant enriched (*p* < 0.05), including melanogenesis, maturity onset diabetes of the young, signaling pathways regulating pluripotency of stem cells, cholinergic synapse, transcriptional misregulation in cancer, retrograde endocannabinoid signaling, and wnt signaling pathway. On the other hand, GO analysis indicated 27 terms enriched (*p* < 0.01) from 336 hypo-methylated genes and 13 terms were showed most significant (*p* < 0.001). KEGG analysis found six pathways were significantly (*p* < 0.05) enriched, including graft-vs.-host disease, autoimmune thyroid disease, staphylococcus aureus infection, asthma, nicotine addiction, and olfactory transduction.

### Identification of Abnormally Methylated Differentially Expressed Genes

**Figure 3:**
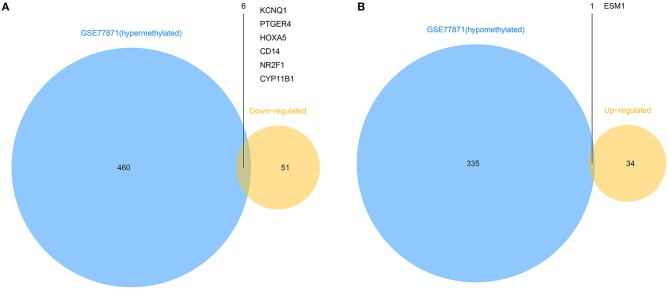
Identification of aberrantly methylated differentially expressed genes. **(A)**. Six hypermethylated and downregulated genes (*KCNQ1, PTGER4, HOXA5, CD14, NR2F1*, and *CYP11B1*) were identified from hypermethylated genes in GSE77871 and common downregulated genes in five mRNA expression profiles. **(B)**. One hypomethylated and upregulated gene (*ESM1*) was identified from hypomethylated genes in GSE77871 and common upregulated genes in five mRNA expression profiles.

To explore the different expressions of those abnormally DMGs, the overlapping of hyper-methylated genes with downregulated genes and hypo-methylated genes with upregulated genes was performed. As shown in Figure 3, this analysis yielded six downregulated hyper-methylated genes and one upregulated hypo-methylated gene.

### Assessment of Seven Genes in TCGA and Oncomine Database

**Figure 4:**
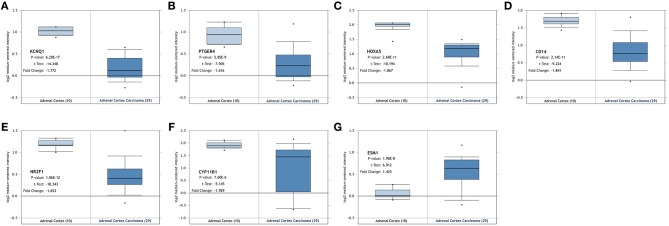
Validation of the identified genes' expression levels in the Oncomine database. Box plot showing the mRNA expression of the seven genes, using data from the Oncomine database. The x-axis shows the number of the normal adrenal cortex samples and adrenal cortex carcinoma samples. The y-axis shows log2 median-centered intensity of gene expression. **(A–G)** is *KCNQ1, PTGER4, HOXA5, CD14, NR2F1, CYP11B1*, and *ESM1*, respectively.

**Figure 5:**
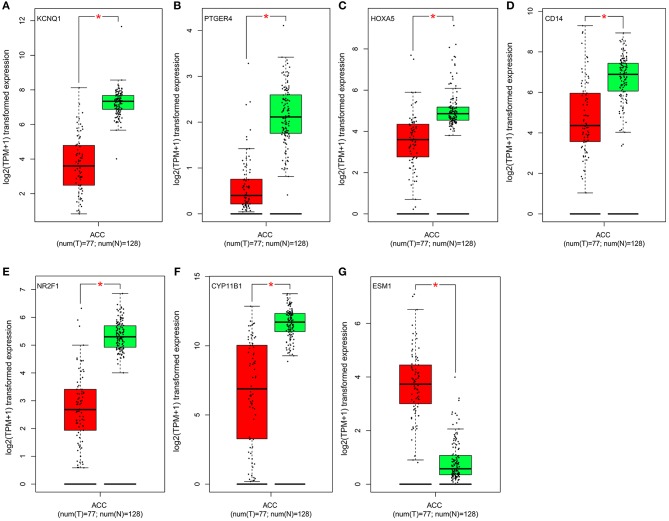
Validation of the identified genes' expression levels in the TCGA database. Box plot showing the expression of seven genes in mRNA, using data from the TCGA database. The x-axis of the plot shows the number of the normal adrenal cortex sample and adrenal cortex carcinoma samples. The y-axis shows the expression data after log2(TPM+1) transformation. A *p* < 0.05 was regarded as significant. **(A–G)** is *KCNQ1, PTGER4, HOXA5, CD14, NR2F1, CYP11B1*, and *ESM1*, respectively.

The seven genes identified above were further validated in the Oncomine database and TCGA database. As shown in Figures 4, 5, significant differences were identified and determined to be identical to those differences found between tumor and normal samples in both databases regarding the expression of the six downregulated hyper-methylated genes and the one upregulated hypo-methylated gene.

### Genetic Information and Methylation Statuses of the Seven Genes in TCGA

**Figure 6:**
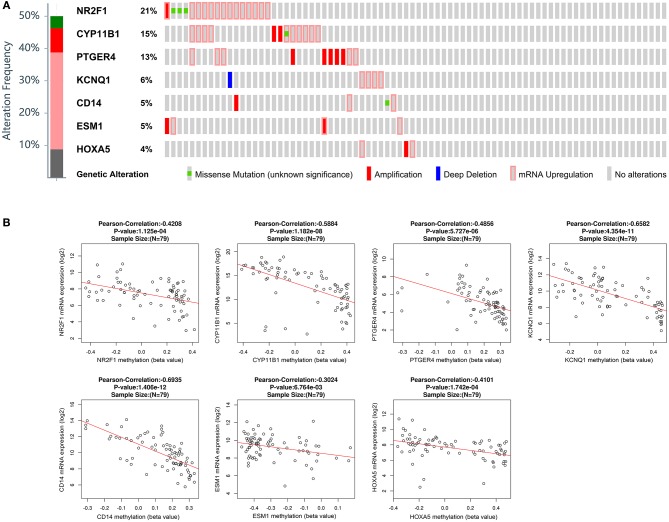
Genetic alterations connected with the identified genes, and the correlation between mRNA expression and DNA methylation in the TCGA database. **(A)** A visual summary across a set of ACCs (data from adrenocortical carcinoma in TCGA, provisional) shows the genetic alteration of the identified genes which were altered in 40 (50.6%) of the 79 patients. **(B)** Correlation between mRNA expression and DNA methylation for the identified genes in TCGA.

Besides methylation, other genic alterations were explored with the software of cBioPortal. As shown in Figure 6A, it was observed that 40 out of the total 79 cases having genic alterations, such as missense, amplification, etc., occurred most often in *PTGER4, CYP11B1*, and *NR2F1* (13, 15, and 21%, respectively). The correlation of seven genes expression with their DNA methylation was shown in Figure 6B. It was noted that the negative correlation of six downregulated hyper-methylated genes were most significant, indicating that methylation might be highly important in gene expression.

### Correlation of Seven Genes Expression and Methylation With Pathological Stages

**Figure 7:**
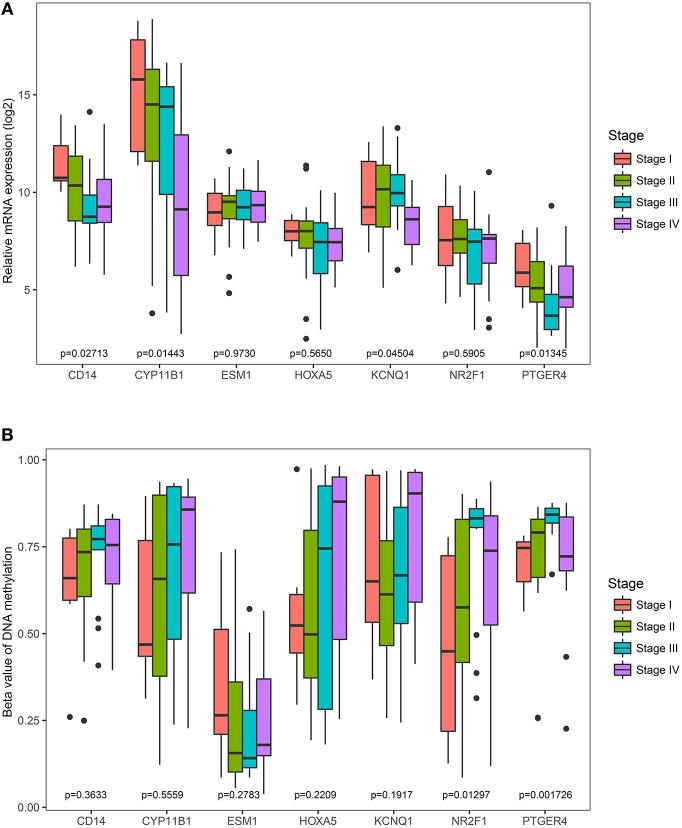
Correlation of the expression levels and methylation levels of identified genes with the pathological stages of ACC. The boxplots show the medians and dispersions of the samples of different pathological stages for each of the seven genes. One-way ANOVA was used for different pathological stages and a *p* < 0.05 was regarded as significant. **(A)** Boxplots of the identified genes transcription level in different pathological stages. **(B)** Boxplots of the identified genes methylation level in different pathological stages.

The Cancer Genome Atlas (TCGA) dataset was used to analyze the association of the identified genes with pathological stage. It was found that the less expressed 4 genes (*CD14, CYP11B1, KCNQ1*, and *PTGER4*) were significantly negatively correlated with pathological stage (Figure 7A). However, the correlation of the rest three genes (*ESM1, HOXA5*, and *NR2F1*) were not significantly different. Comparisons were also made among all genes' methylation status with various pathological stages and it was noted only the hyper-methylated two genes (*PTGER4* and *NR2F1*) were significantly positively correlated with pathological stage (Figure 7B).

### Survival Analysis

**Figure 8:**
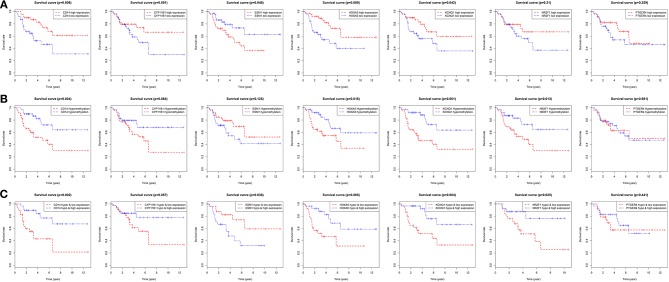
Overall survival analyses of the identified genes in the TCGA dataset. **(A)** Survival curves for patients grouped by gene expression. Red lines represent high expression of the identified genes, while blue lines represent low expression of the identified genes. **(B)** Survival curves for patients grouped by gene methylation. Red lines represent hypermethylation of the identified genes, while blue lines represent hypomethylation of the identified genes. **(C)** Survival curves for patients grouped by gene expression and methylation. Red lines represent hypermethylation and low expression of the identified genes, while blue lines represent hypomethylation and high expression of the identified genes.

On the basis of the clinical information, and data of RNA sequencing and methylation in TCGA, patients were classified in two different categories in accordance to each gene's median expressions. Then the Kaplan-Meier survival curve was plotted. It revealed that the overall rates of survival for the patients with four hyper-methylated genes (*CD14, HOXA5, KCNQ1*, and *NR2F1*), one more expressed gene (*ESM1*) or three less expressed genes (*CD14, HOXA5*, and *KCNQ1*) were greatly lower, which were shown in Figures 8A,B. When their expression and methylation were combined, the overall rates of survival significantly negatively correlated with hypermethylation and low expression of five genes (*CD14, HOXA5, KCNQ1*, and *NR2F1*) along with hypomethylation and high expression of *ESM1* (Figure 8C).

### Validation of the Methylation Status of Six Genes in GSE49280

**Figure 9:**
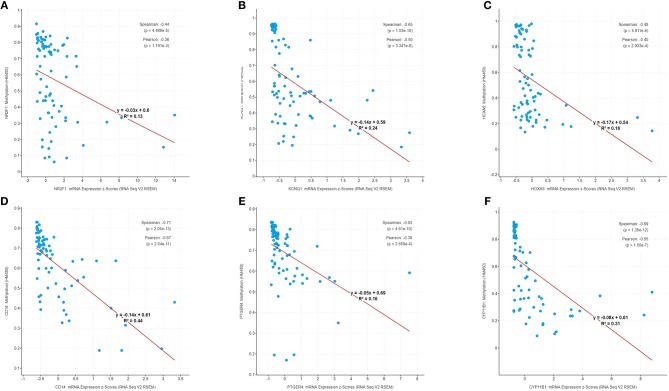
Correlation of methylation and expression of six genes in GSE49280 dataset. The linear regression shows the correlation between mRNA expression and DNA methylation for the identified genes in GSE49280. Spearman and Pearson correlation coefficient were used for different pathological stages and a *p* < 0.05 was regarded as significant. **(A–F)** is *NR2F1, KCNQ1, HOXA5, CD14, PTGER4*, and *CYP11B1*, respectively.

The correlation of six genes expression with their DNA methylation was shown in Figure 9. It was noted that the negative correlation of six downregulated hyper-methylated genes were most significant, indicating that methylation might be highly important in gene expression.

### Validation of the Methylation Status of Six Genes in ACC Tissues and Cells

**Figure 10:**
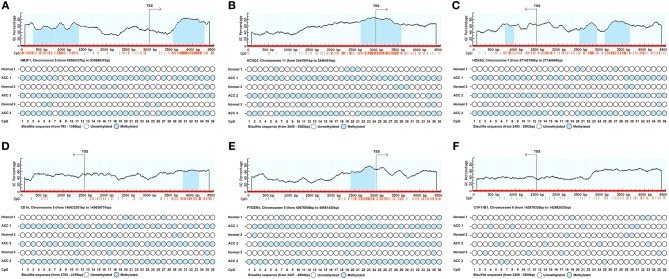
Schematic representation and bisulfite sequence analyses of six identified hypermethylated genes' promoter region. Upper panels show sequence features of six genes such as GC percent (y-axis), CpG island (blue area) and CpG site (orange vertical line) from TSS (transcription start site). Lower panels show the bisulfite sequence analyses of 36 CpG sites of six genes' promotor regions in ACC tissues and adjacent normal ones. **(A–F)** is *NR2F1, KCNQ1, HOXA5, CD14, PTGER4*, and *CYP11B1*, respectively.

**Figure 11:**
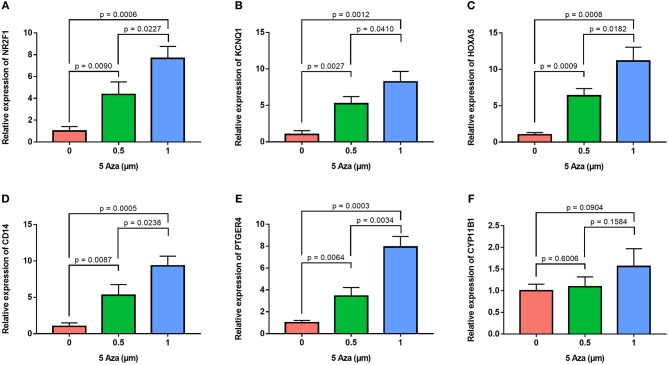
Validation of methylation of six genes in SW13 cells. Real time RT-PCR analysis detected the mRNA levels of six genes in SW13 cells after the treatment by the demethylating agent 5-Aza at 0, 0.5, and 1 μm for 48 h. **(A–F)** is *NR2F1, KCNQ1, HOXA5, CD14, PTGER4*, and *CYP11B1*, respectively.

**Figure 12:**
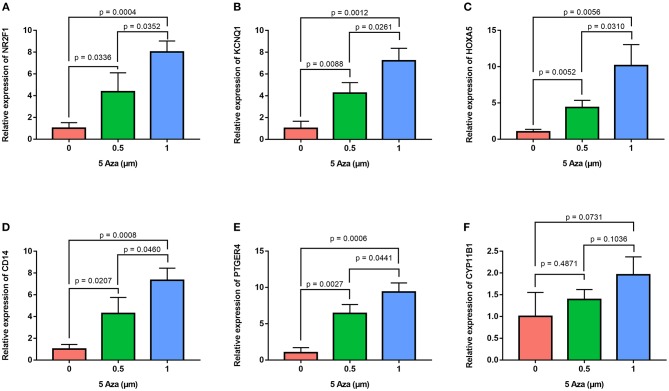
Validation of methylation of six genes in NCI-H295R cells. Real time RT-PCR analysis detected the mRNA levels of six genes in NCI-H295R cells after the treatment by the demethylating agent 5-Aza at 0, 0.5, and 1 μm for 48 h. **(A–F)** is *NR2F1, KCNQ1, HOXA5, CD14, PTGER4*, and *CYP11B1*, respectively.

In order to confirm the methylation of the six hyper-methylated gene promoters in ACC, bisulfite sequence analyses were performed on three ACC samples and three adjacent normal ones, demonstrating that methylation of all CpG sites of five gene promoters were more frequent in ACC rather than in normal samples (Figures 10A–E). Among them, the degree of methylation of *CYP11B1* is low and there is no significant difference compared with normal tissues (Figure 10F). Next, to further investigate the relationship between methylation and expression of six genes, SW13 and NCI-H295R cells were cultured with the demethylating agent 5-Aza-2′-deoxycytidine, and quantitative real time RT-PCR indicated the restoration of seven genes' expression in ACC cells (Figures 11A–F, 12A–F).

### Validation of the mRNA Expression of Seven Genes in ACC and Normal Tissues

**Figure 13:**
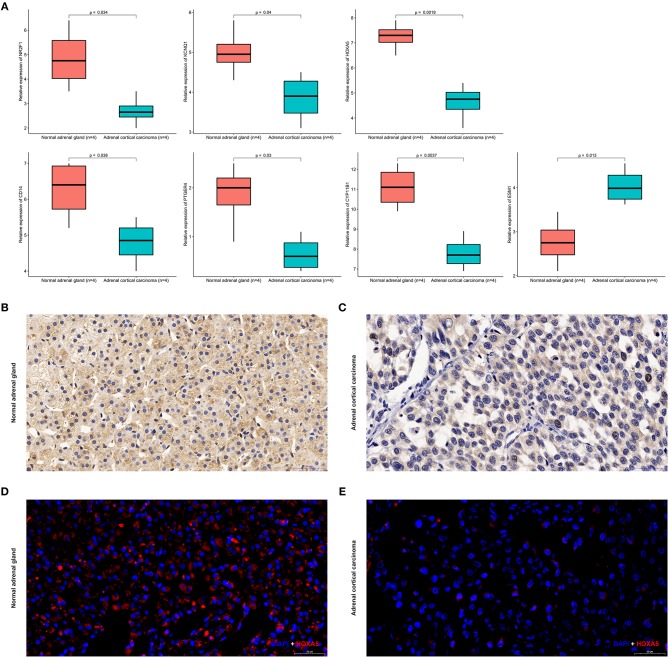
Expression of seven identified genes in normal adrenal tissues and adrenocortical carcinoma tissues. **(A)** Transcriptional levels of seven genes in ACC tissues and normal ones. **(B)** Immunohistochemistry of *HOXA5* in normal samples. The magnification is × 200. **(C)** Immunohistochemistry of *HOXA5* in ACC tissues. The magnification is × 200. **(D)** Immunofluorescence of *HOXA5* in normal samples. Cy3-immunofluorescence (red) indicates *HOXA5* was observed in normal adrenal cortex. DAPI (blue) indicates nuclear staining in normal adrenal gland tissue. The magnification is × 200. **(E)** Immunofluorescence of *HOXA5* in ACC samples. Cy3-immunofluorescence (red) indicates *HOXA5* was observed in ACC tissue. DAPI (blue) indicates nuclear staining in normal adrenal gland tissue. The magnification is × 200.

Expression of *CYP11B1, PTGER4, HOXA5, CD14, NR2F1, KCNQ1*, and *ESM1* mRNA were determined using quantitative real time RT-PCR between ACC samples and normal ones. *HOXA5* expression was most significantly downregulated at the transcription level (*p* = 0.0019) in ACC. Real time RT-PCR also showed that for the other six genes that *NR2F1* (*p* = 0.034), *KCNQ1* (*p* = 0.04), *CD14* (*p* = 0.038), *PTGER4* (*p* = 0.03), *CY11B1* (*p* = 0.0037), and *ESM1* (*p* = 0.0013) the mRNA expression was significantly altered in the ACC samples (Figure 13A).

### Immunohistochemistry and Immunofluorescence Analysis of HOXA5 in ACC and Normal Tissues

As shown in Figures 13B–E, *HOXA5* is localized in adrenal cortical cytoplasm and nucleus. In normal adrenal gland, *HOXA5* was mainly present in adrenocortical cells nuclei and partly present in the adrenocortical cytoplasm. In ACC, the localization is similarly with that in normal ones, but in ACC, the fluorescence intensity is significantly lower than that of normal. That is to say, immunohistochemistry and immunofluorescence also confirm that the expression of the hypermethylated gene *HOXA5* in ACC is lower than that in normal tissues.

## Discussion

The current study, for the first time, identified six downregulated hyper-methylated genes and one upregulated hypo-methylated gene related to ACC. Additionally, these genes are found to be correlated with different pathological stages and overall rate of survival of ACCs. Our study suggests these abnormally methylated and deregulated genes could be used as therapeutic targets or biomarkers for ACC patients to be precisely diagnosed and effectively treated.

The six downregulated hyper-methylated genes were *CYP11B1, PTGER4, HOXA5, CD14, NR2F1*, and *KCNQ1* and the one upregulated hypo-methylated gene was *ESM1*. These abnormal expressions of these genes were further confirmed in other databases of TCGA and Oncomine. It is well-known that DNA methylation can turn off the activity of certain genes, while demethylation induces gene reactivation and expression. Therefore, hyper-methylation could lead to gene downregulation, functionally similar to a tumor suppressor gene, while hypo-methylation could result in gene upregulation, functionally similar to oncogenes. Thus, the hyper-methylated upregulated genes and hypo-methylated downregulated genes were not further analyzed in the present study.

It is widely acknowledged that the imbalance between oncogene and tumor suppressor genes plays a key role in tumorigenesis. Dysregulation of genes identified in our study may be involved in the development of ACC. Indeed, these seven genes have unique functions. As an enzyme coming from the big family of the cytochrome P450 genes, *CYP11B1* can serve as a catalyzer for lots of drug metabolism reactions, as well as the synthesis of lipids like steroids and cholesterol. Studies have found the association of downregulated members from cytochrome P450 with the incidence of various cancers, such as renal, breast, and colorectal cancers (31). *PTGER4* is from the family of G protein coupled receptors, which resides on 5p13.1, encoding one out of the four identified receptors for prostaglandin E2 (32, 33). *HOXA5* (homeobox A5) is a member of highly-conserved HOX gene family that is responsible for controlling the differentiation of cells and the development of the embryo (34). *CD14* (CD14 molecule) is one of the critical elements that compose the immune system of humans and it has certain functions in protecting human bodies from the microbial products in the environment (35). *NR2F1* is a nuclear hormone receptor and transcriptional regulator (36–38). *KCNQ1* is a voltage-gated potassium channel required for the repolarization phase of the cardiac action potential (39). Additionally, the upregulated hypo-methylated gene *ESM1*, encodes one secreted protein, whose expression mainly occurs within the endothelial cells of human kidneys and lungs (40). Although the specific functions of these seven genes in ACC have never been demonstrated, their mutation and expression dysregulation may have impacts on tumorigenesis, staging, and prognosis of ACC.

The current study also demonstrated a negative correlation between gene expression and pathologic stages. However, only the four less expressed genes (*CYP11B1, PTGER4, CD14, KCNQ1*) identified in the study were significantly associated with a higher pathology stage. Consistently, our study showed hypermethylation of two genes (*PTGER4* and *NR2F1*) led to a higher pathology stage. Finally, five genes (*CYP11B1, PTGER4, CD14, KCNQ1*, and *NR2F1*) were significantly correlated with pathology stages. These genes functionally act as tumor suppressor genes when the genes are hypermethylated. In survival analysis, the overall rates of survival for the patients with 4 hyper-methylated genes (*CD14, HOXA5, KCNQ1*, and *NR2F1*), one more expressed gene (*ESM1*) or three less expressed genes (*CD14, HOXA5*, and *KCNQ1*) were greatly lower. When their expression and methylation were combined, the overall rates of survival significantly negatively correlated with hypermethylation and low expression of five genes (*CD14, HOXA5, KCNQ1*, and *NR2F1*) along with hypomethylation and high expression of *ESM1*. In addition, altered DNA methylation patterns have been demonstrated to be the first detectable neoplastic changes associated with tumorigenesis. Furthermore, these DNA changes may be easily detected in the plasma or serum of cancer patients, thereby highlighting the potential of DNA methylation as a novel non-invasive molecular marker for cancer diagnosis and prognosis (41, 42). Kim et al. showed the rate of 3 year survival of the patients with acute myelocytic leukemia depends on their levels of *HOXA5* methylation (43). In our study, survival analysis results demonstrated that *HOXA5* was closely related with overall survival, indicating that *HOXA5* could be a predictor of poor prognosis in ACC patients. In addition, compared to the patients with lesser expression of *ESM1*, those with highly expressed *ESM1* were reported to have an obviously worse relapse-free survival in triple-negative breast cancer (44). In our study, we found that *ESM1* was hypomethylated and upregulated in ACCs, indicating that aberrant methylation in ACCs might lead to the upregulation of *ESM1*, resulting in ACC tumorigenesis. In addition, some other genes like *KCNQ1, CD14*, and *NR2F1* were significantly related with overall survival, which might be able to be used as a prognosis indicator for ACC. Besides the methylation, other genetic alterations, such as missense mutation, amplification and deep deletion could also contribute to tumorogenesis. Therefore, not all hypermethylated genes identified in the present study are indeed associated with a higher pathological stage or a poorer survival.

Our quantitative real-time PCR and sodium bisulfite sequencing data strongly indicates that five genes (*NR2F1, KCNQ1, HOXA5, CD14*, and *PTGER4*) undergo methylation induced regulation of gene expression in ACC tissues. ACC tissues have a higher number of methylated sites of 36 CpG sites as compared to the normal samples. In contrast, expression of these five genes in normal adrenal tissues were higher than that in ACC tissues. We found that the degree of methylation of the *CYP11B1* gene is inconsistent with our prediction, but its low expression is consistent with our prediction. Because of the multiple ways to inhibit gene expression, this difference may be due to other pathways leading to low expression of *CYP11B1* in ACC. However, we have noticed that hypermethylation of the *CYP11B1* gene and the loss of its expression may be related to the occurrence of hypercortisolemia (45). Interestingly, among the seven genes we screened, *CYP11B1* and *HOXA5* are also among the adrenal specific expression of the gene signature defined by Zheng et al. (46). In addition, as Rechache et al. discovered, we also screened out *KCNQ1* as one of the genes that are hypermethylated and downregulated in ACC (47). We included the dataset GSE49280 (48) in our research and found that the negative correlation of six downregulated hyper-methylated genes were significant, indicating that methylation might be highly important in gene expression. In order to further explore the expression and localization of *HOXA5* gene, we performed novel immunohistochemistry and immunofluorescence experiments in ACC and adjacent normal tissues, and found that the gene is mainly expressed in the nucleus.

In conclusion, we identified seven genes hypo/hyper methylated and dysregulated, which could play important roles in the development and progression of ACCs. Additionally, some genes were found to negatively correlate with different pathological stages or overall rates of survival of ACCs. We have verified these seven genes by various experimentation and have demonstrated that they are differentially expressed in ACC. Among six hypermethylated genes we identified in screening, five of them (*NR2F1, KCNQ1, HOXA5, CD14, PTGER4*) were found hypermethylated through BSP experiments. Our novel data suggests these abnormally methylated genes could be used as therapeutic targets and biomarkers for ACC patients to be precisely diagnosed and effectively treated. However, further studies are required to validate the functional activities and molecular mechanism of these genes on ACC.

## Ethics Statement

The use of these ACC specimens was approved by the Ethics Committee at Zhongnan Hospital of Wuhan University, and informed consent was obtained from all patients.

## Author Contributions

HX, PC, and XZ conceived and designed the study. HX, XW, and WH performed the analysis procedures. ZL, GZ, and DX analyzed the results. HX, PC, MH, MD, and XZ contributed to the writing of the manuscript. All authors reviewed the manuscript.

### Conflict of Interest Statement

The authors declare that the research was conducted in the absence of any commercial or financial relationships that could be construed as a potential conflict of interest.
